# Co-infection with macrolide-resistant and macrolide-susceptible *Bordetella pertussis* strains in an infant: a case report

**DOI:** 10.1128/asmcr.00242-25

**Published:** 2026-04-24

**Authors:** Kentaro Koide, Kaoru Ikeda, Kohei Osada, Yoshio Sakurai, Masataka Goto, Tsuyoshi Kenri, Nao Otsuka

**Affiliations:** 1 Department of Bacteriology II, National Institute of Infectious Diseases, Japan Institute for Health Security 739298 , Musashimurayama, Tokyo, Japan; 2 Department of Pediatrics, National Defense Medical College 13077, Tokorozawa, Saitama, Japan; 3 Department of Pediatric Critical Care Medicine, Saitama Medical Center, Saitama Medical University 13031, Kawagoe, Japan; Rush University Medical Center, Chicago, Illinois, USA

**Keywords:** *Bordetella pertussis*, macrolide resistance, co-infection

## Abstract

**Background:**

*Bordetella pertussis* infection causes severe respiratory illness, particularly in young infants. Although macrolides are recommended as the first-line treatment, macrolide-resistant strains have emerged in several countries. In Japan, a nationwide pertussis epidemic occurred in 2025, during which numerous infections were reported not only due to macrolide-susceptible *B. pertussis* (MSBP) but also due to macrolide-resistant strains (MRBP).

**Case Summary:**

A 2-month-old female developed a persistent cough and was admitted to the pediatric intensive care unit (PICU), where she received frequent suctioning and short-term high-flow nasal cannula therapy. BioFire FilmArray Respiratory Panel 2.1 analysis of her nasal swab detected *B. pertussis* DNA; azithromycin was therefore administered for 5 days. Direct sequencing of the DNA extracted from her nasal swabs initially indicated an A2047G mutation in the 23S rRNA gene of *B. pertussis,* indicating MRBP infection, although the electropherogram was unclear. Trimethoprim-sulfamethoxazole was initiated on day 8 of her PICU stay following confirmation of MRBP infection. Subsequently, bacterial culture yielded two different *B. pertussis* strains, which were confirmed as MRBP and MSBP through antimicrobial susceptibility testing. Multilocus variable-number tandem-repeat analysis and whole-genome analysis demonstrated that the strains were genetically unrelated. The patient was discharged and subsequently fully recovered after a 14 day course of trimethoprim-sulfamethoxazole.

**Conclusion:**

To our knowledge, this is the first confirmed case of co-infection with MRBP and MSBP strains. Such mixed infections may be missed by the diagnostic methods currently used in clinical practice, highlighting the importance of careful interpretation of test results.

## INTRODUCTION

*Bordetella pertussis* is a causative agent of pertussis. Although this highly contagious respiratory pathogen can affect all age groups, infants are at the highest risk of severe disease. Macrolides such as azithromycin remain the first-line antibiotics for treatment; however, macrolide-resistant *B. pertussis* (MRBP) has been reported in several countries over the past two decades (1, 2). Notably, in China, nearly 90% of recent pertussis cases have been attributed to MRBP (3). Thus, the emergence of MRBP is recognized as a growing public health concern.

Here, we report a case of co-infection with MRBP and macrolide-susceptible *B. pertussis* (MSBP) strains in a patient with pertussis. In Japan, after several years of low incidence since 2020, pertussis cases began to rise in mid-2024 and culminated in a nationwide epidemic in 2025. During this epidemic, numerous cases caused by either MSBP or MRBP alone were reported (4–6). To our knowledge, this is the first documented case of simultaneous infection with both MRBP and MSBP strains in a single patient. This report describes the patient’s clinical course and the genomic characteristics of the two strains.

## CASE PRESENTATION

The patient was a female infant younger than 2 months of age with no underlying medical conditions. She had not yet received pertussis vaccination because, according to the current immunization schedule in Japan, routine pertussis immunization begins at 2 months of age and consists of a four-dose series of the pentavalent diphtheria-tetanus-acellular pertussis-inactivated poliovirus*-Haemophilus influenzae* type b (DTaP-IPV-Hib) vaccine. Around the time she developed pertussis symptoms, several family members (her father, mother, and 8-year-old brother) also experienced respiratory symptoms. Although no diagnostic testing was performed for them, intrafamilial transmission was considered the most likely source of infection.

**Fig 1:**
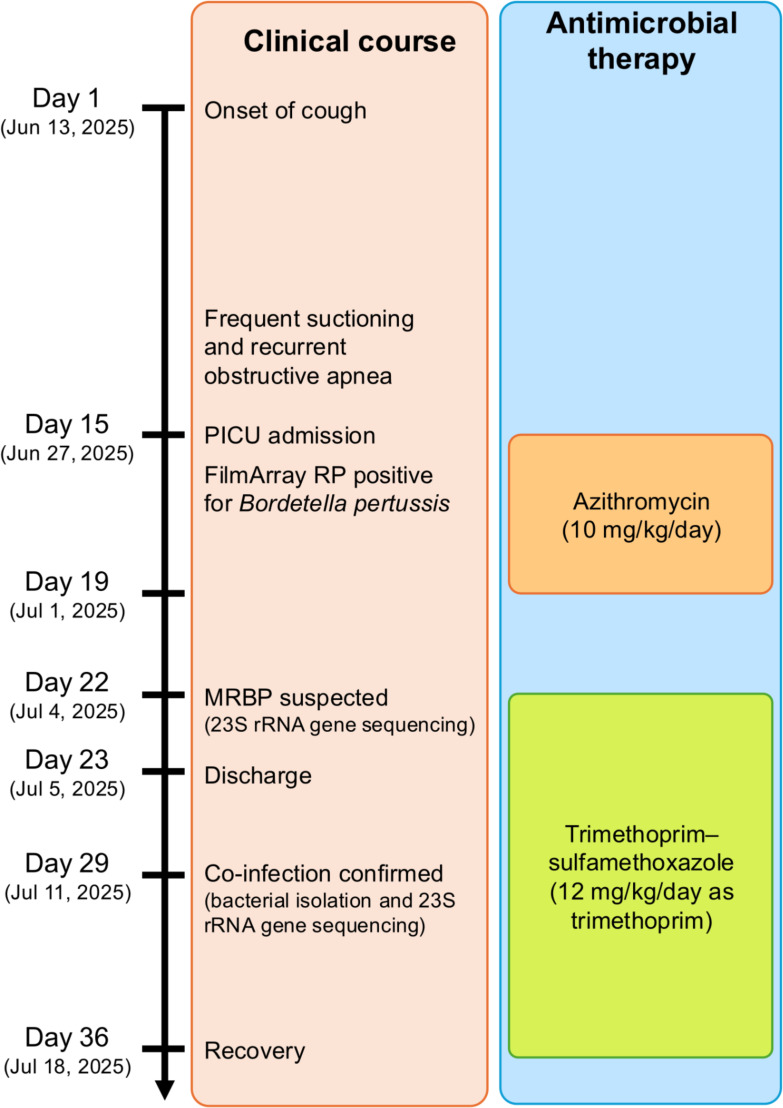
Timeline of the clinical course and antimicrobial therapy in the present case.

The patient’s cough began when she was 50 days old and was initially managed with symptomatic treatment at a local clinic and a secondary hospital. Although her general condition was stable, she had copious airway secretions requiring frequent suctioning and recurrent episodes of obstructive apnea with oxygen desaturation during coughing. Given these symptoms and her young age, she was transferred to the pediatric intensive care unit (PICU) at Saitama Medical Center in Kawagoe, Japan, on day 15 after symptom onset. At PICU admission, the cough persisted and her body temperature was 37.6°C. Laboratory testing showed a white blood cell count of 15,300 cells/μL (reference range for infants, approximately 5,000–19,500 cells/μL), with neutrophils accounting for 47.6% and lymphocytes for 45.6%. Chest radiography showed no evidence of pneumonia. A nasal swab obtained from the patient was subjected to FilmArray Respiratory Panel 2.1 (BioFire Diagnostics, Salt Lake City, UT) testing; *B. pertussis* was detected. Azithromycin (10 mg/kg/day) was administered for the first 5 days of the PICU stay, and trimethoprim-sulfamethoxazole (12 mg/kg/day, calculated based on trimethoprim) was started on day 8 of hospitalization (day 22 after symptom onset). By the time trimethoprim-sulfamethoxazole was initiated, her respiratory status had stabilized, and she was discharged the following day. The medication was continued after discharge for 14 days. At the outpatient visit two weeks later, her cough had completely resolved. The overall clinical course, including key diagnostic findings and antimicrobial treatments, is summarized in Fig. 1.

**Fig 2:**
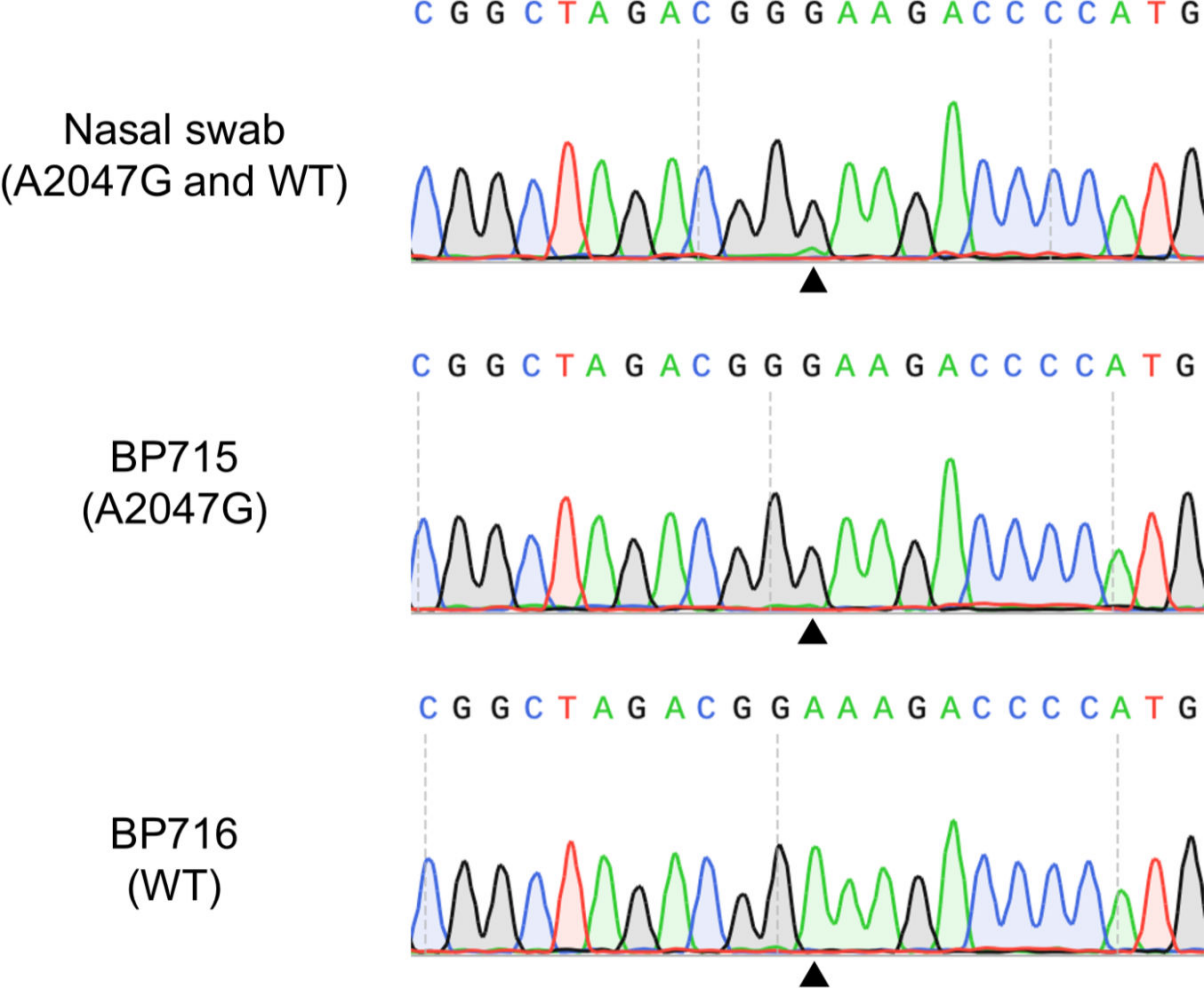
Sanger sequencing chromatograms of the 23S rRNA gene region surrounding the A2047G mutation. Chromatograms display sequences obtained from DNA extracted directly from the nasal swab (top), the macrolide-resistant strain BP715 (middle), and the macrolide-susceptible strain BP716 (bottom). The nucleotide position corresponding to the A2047G mutation is indicated by arrowheads.

At PICU admission, two additional nasal swabs were collected and sent to the National Institute of Infectious Diseases (NIID) for testing for macrolide resistance in *B. pertussis*. DNA was extracted directly from the swab specimens using the QIAamp DNA Micro Kit (Qiagen, Hilden, Germany). Sanger sequencing of the 23S rRNA gene was then performed as previously described (7). The chromatogram showed a predominant “G” peak at position 2047, corresponding to the A2047G mutation associated with macrolide resistance, along with a minor wild-type “A” peak (Fig. 2). Accordingly, MRBP infection was suspected, and trimethoprim-sulfamethoxazole therapy was subsequently initiated.

Additionally, bacterial isolation was performed at NIID and yielded two *B. pertussis* strains with different macrolide susceptibility profiles. Swab specimens collected for molecular testing were streaked onto Bordetella CFDN agar (Nikken-bio, Kyoto, Japan) and incubated at 36°C for 7 days, yielding 12 colonies. Each colony was subcultured, and DNA was extracted using the boiling method. All colonies were identified as *B. pertussis* by detection of the *IS481* insertion sequence using a real-time PCR assay (8). Sanger sequencing of DNA showed that nine colonies carried the A2047G mutation and three retained the wild-type allele; these representative colonies were designated BP715 and BP716, respectively. Antimicrobial susceptibility testing using Etest (bioMérieux, Craponne, France) was performed as previously described (9) and confirmed high-level macrolide resistance in BP715 (minimum inhibitory concentration >256 µg/mL for erythromycin, azithromycin, and clarithromycin), whereas BP716 was macrolide-susceptible (Table 1).

**TABLE 1:** Minimum inhibitory concentrations (MICs) for BP715 and BP716

Antimicrobials	MICs (μg/mL)	
	BP715	BP716
Erythromycin	>256	0.023
Clarithromycin	>256	0.023
Azithromycin	>256	0.023
Trimethoprim/sulfamethoxazole	0.125	0.125
Piperacillin	≤0.016	≤0.016
Ampicillin	0.125	0.094
Minocycline	0.047	0.064
Levofloxacin	0.012	0.012
Gentamicin	0.500	0.500

**Fig 3:**
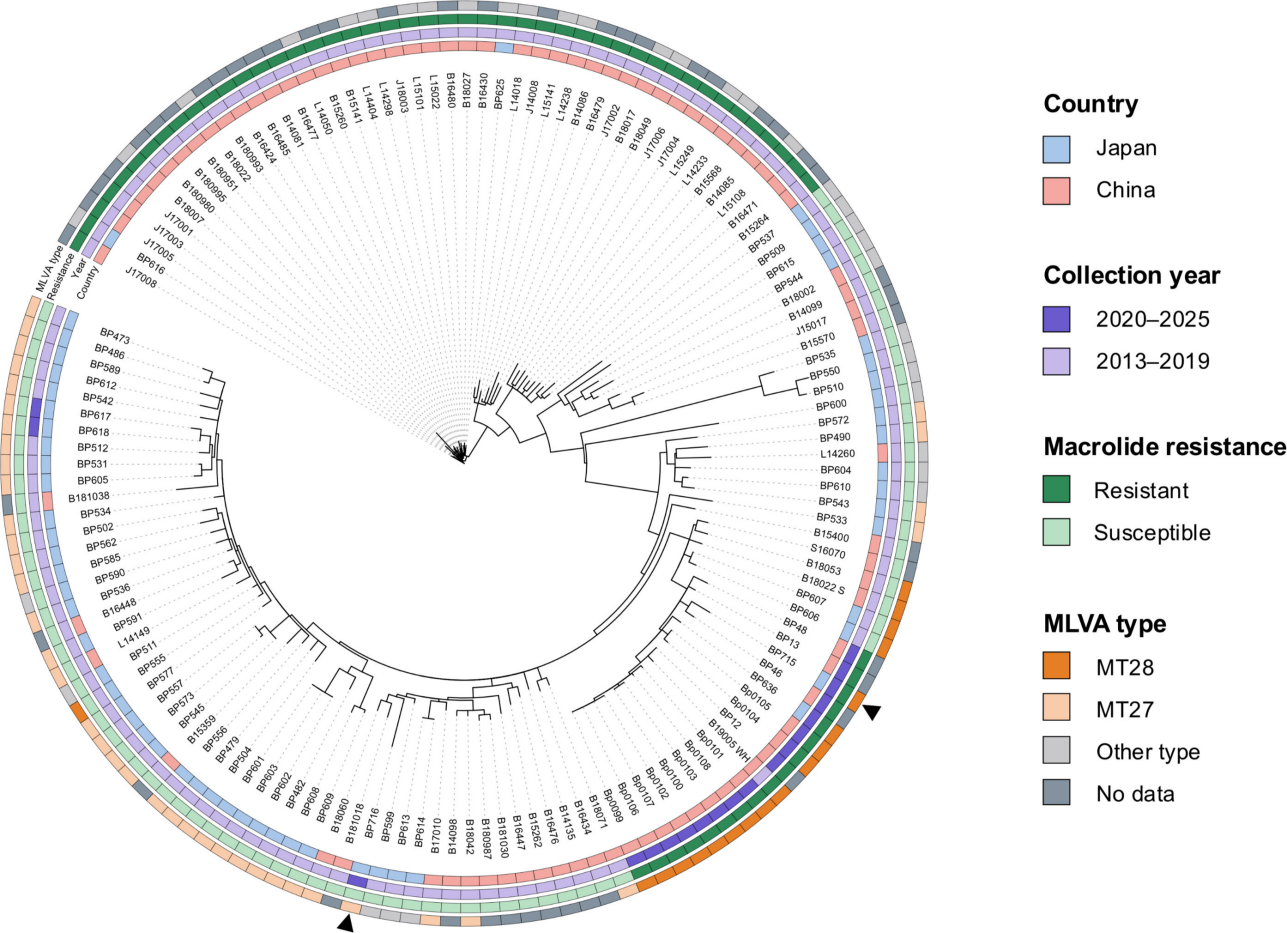
A phylogenetic tree based on single nucleotide variants of *Bordetella pertussis* isolates from Japan and China. Black arrowheads indicate the positions of BP715 and BP716.

Genomic analyses showed that BP715 and BP716 were unrelated *B. pertussis* strains. Their genetic relationship was evaluated using multilocus variable-number tandem-repeat analysis (MLVA) and whole-genome sequencing, as described previously (9, 10). MLVA assigned BP715 to MT28 and BP716 to MT27, indicating distinct genotypes. A phylogenetic tree of single-nucleotide variants placed the two isolates in separate clades (Fig. 3), incorporating both genomic data generated in this study and previously reported isolates (4, 9, 11–16). BP715 was clustered within the MT28-MRBP clade, which predominantly comprises MRBP isolates collected after 2020, whereas BP716 was positioned in a genetically distinct clade containing MSBP isolates circulating prior to 2019. Whole-genome analysis further supported the Sanger sequencing results. The A2047G mutation was detected in all three copies of the 23S rRNA gene in BP715, while BP716 retained the wild-type allele. These findings indicate that the two strains came from different circulating populations, supporting the hypothesis that the resistant strain did not emerge from the susceptible strain within the host but instead represented simultaneous infection with two genetically distinct strains.

## DISCUSSION

This case describes a pertussis infection in which both MRBP and MSBP strains were isolated from a single patient. The patient showed typical clinical features of pertussis and recovered without complications. Molecular testing of DNA extracted from the nasal swab revealed mixed “A” and “G” peaks at the A2047G site, initially causing diagnostic uncertainty. Subsequent bacterial culture and antimicrobial susceptibility testing confirmed the coexistence of macrolide-resistant and -susceptible strains; whole-genome sequencing demonstrated that the two isolates were genetically unrelated, indicating true co-infection rather than the emergence of resistance during treatment. These findings highlight an underrecognized infection pattern and illustrate the diagnostic and public health challenges associated with the increasing prevalence of MRBP.

No clear association between disease severity and co-infection was identified in this case. Although co-infection with a resistant strain could theoretically reduce responsiveness to antimicrobial therapy and thereby worsen the clinical course, its contribution to the need for intensive care could not be assessed because antimicrobial treatment was only initiated after PICU admission. In contrast, host factors and delayed initiation of therapy offer a more plausible explanation for the progression to severe disease. Severe pertussis predominantly affects infants younger than 3 months and those who have not yet received pertussis vaccination (17); this patient met both of these major risk factors. Furthermore, the patient’s symptoms were initially managed under observation, and antimicrobial therapy was not initiated until 2 weeks after symptom onset. The late initiation of antimicrobial treatment in this case raises the possibility that the disease may have advanced during the untreated period. Taken together, these considerations suggest that, rather than co-infection with MRBP and MSBP, host vulnerability and delayed treatment were the primary factors associated with disease severity.

Accurately identifying co-infection with MRBP and MSBP remains difficult using current diagnostic methods. Macrolide resistance is typically assessed either by antimicrobial susceptibility testing of cultured isolates or by detecting the A2047G mutation in the 23S rRNA gene. Successful isolation of *B. pertussis* from clinical specimens is often difficult. Even when colonies are obtained, their number is usually small, increasing the likelihood that only one strain will be detected while others are missed. Molecular testing can identify the A2047G mutation in both cultured isolates and directly from clinical specimens, enabling faster assessment of macrolide resistance. However, when one strain is present at a much higher abundance, the signal from the less abundant strain may fall below the detection threshold. Additionally, the presence of both “A” and “G” peaks at the A2047G position does not necessarily indicate co-infection; mixed signals may also arise from other bacterial species with similar 23S rRNA sequences, such as *Achromobacter* spp. The reliability of currently available diagnostic methods for detecting co-infection thus remains limited.

The potential overlooking of MRBP infections represents an important public health concern. When MRBP is not accurately detected, patients may continue to receive macrolide therapy with limited efficacy, prolonging infectiousness and increasing the risk of transmission within the community. Additionally, if many cases are caused by undetected co-infection, the true prevalence of MRBP may be underestimated. Such underestimation could obscure the actual epidemiological situation and delay appropriate public health responses to emerging resistant lineages. To mitigate these risks, awareness of possible co-infection among clinicians, microbiology laboratories, and public health authorities involved in pertussis surveillance and control, and improved diagnostic approaches are essential.

### Conclusion

To our knowledge, this report describes the first documented case of co-infection with MRBP and MSBP strains in a single patient. Genomic analyses demonstrated that the two isolates were genetically distinct, indicating infection with two independent lineages. Although the mixed infection did not appear to influence clinical severity in this case, the coexistence of MRBP and MSBP highlights significant diagnostic challenges. Current methods for detecting macrolide resistance may fail to identify MRBP when susceptible strains predominate, leading to underestimation of resistance prevalence and inappropriate antimicrobial therapy. With the ongoing spread of MRBP, awareness of possible co-infection is essential for accurate diagnosis, treatment selection, and surveillance.

## Data Availability

Sequencing reads obtained in this study are available in the DDBJ Sequence Read Archive under BioProject PRJDB7578, with run accession numbers DRR795565 (BP715) and DRR795566 (BP716).

## References

[1] Ivaska L, Barkoff AM, Mertsola J, He Q. 2022. Macrolide resistance in Bordetella pertussis: current situation and future challenges. Antibiotics (Basel) 11:1570. doi:10.3390/antibiotics11111570 36358225 PMC9686491

[2] Rodrigues C, Bouchez V, Soares A, Trombert-Paolantoni S, Aït El Belghiti F, Cohen JF, Armatys N, Landier A, Blanchot T, Hervo M, Toubiana J, Brisse S, REMICOQ study group. 2024. Resurgence of Bordetella pertussis, including one macrolide-resistant isolate, France, 2024. Euro Surveill 29:2400459. doi:10.2807/1560-7917.ES.2024.29.31.2400459 39092529 PMC11295439

[3] Li L, Deng J, Ma X, Zhou K, Meng Q, Yuan L, Shi W, Wang Q, Li Y, Yao K. 2019. High prevalence of macrolide-resistant Bordetella pertussis and ptxP1 genotype, Mainland China, 2014-2016. Emerg Infect Dis 25:2205–2214. doi:10.3201/eid2512.181836 31742507 PMC6874251

[4] Iwasaki T, Koide K, Kido T, Nakagawa S, Goto M, Kenri T, Suzuki H, Otsuka N, Takada H. 2025. Fatal case of macrolide-resistant Bordetella pertussis infection, Japan, 2024. J Infect Chemother 31:102727. doi:10.1016/j.jiac.2025.102727 40348379

[5] Tsukahara H, Araki K, Cho Y, Fujiwara N, Matsuoka T, Kakita T, Morichika S, Ohnishi M. 2025. Severe macrolide-resistant Bordetella pertussis (ptxP3) infection in Japanese infants: first report of cases requiring intensive care. Int J Infect Dis 158:107960. doi:10.1016/j.ijid.2025.107960 40541771

[6] Kakita T, Taira H, Kudeken T, Kuniyoshi M, Takara T, Teruya M, Kyan H, Ohnishi M. 2025. Multiclonal dissemination of ptxP3 allele macrolide-resistant Bordetella pertussis in Okinawa, Japan. Jpn J Infect Dis. doi:10.7883/yoken.JJID.2025.123 40887273

[7] Wang Z, Cui Z, Li Y, Hou T, Liu X, Xi Y, Liu Y, Li H, He Q. 2014. High prevalence of erythromycin-resistant Bordetella pertussis in Xi’an, China. Clin Microbiol Infect 20:O825–30. doi:10.1111/1469-0691.12671 24816168

[8] Kamachi K, Yoshino S, Katsukawa C, Otsuka N, Hiramatsu Y, Shibayama K. 2015. Laboratory-based surveillance of pertussis using multitarget real-time PCR in Japan: evidence for Bordetella pertussis infection in preteens and teens. New Microbes New Infect 8:70–74. doi:10.1016/j.nmni.2015.10.001 27076914 PMC4815931

[9] Koide K, Uchitani Y, Yamaguchi T, Otsuka N, Goto M, Kenri T, Kamachi K. 2024. Whole-genome comparison of two same-genotype macrolide-resistant Bordetella pertussis isolates collected in Japan. PLoS One 19:e0298147. doi:10.1371/journal.pone.0298147 38359004 PMC10868825

[10] Koide K, Yao S, Chiang CS, Thuy PTB, Nga DTT, Huong DT, Dien TM, Vichit O, Vutthikol Y, Sovannara S, Samnang C, Takayama I, Ainai A, Nakajima N, Otsuka N, Kamachi K, Saitoh A. 2022. Genotyping and macrolide-resistant mutation of Bordetella pertussis in East and South-East Asia. J Glob Antimicrob Resist 31:263–269. doi:10.1016/j.jgar.2022.10.007 36270447 PMC9750937

[11] Wu X, Du Q, Li D, Yuan L, Meng Q, Fu Z, Xu H, Yao K, Zhao R. 2022. A cross-sectional study revealing the emergence of erythromycin-resistant Bordetella pertussis carrying ptxP3 alleles in China. Front Microbiol 13:901617. doi:10.3389/fmicb.2022.901617 35923401 PMC9342848

[12] Cai J, Chen M, Liu Q, Luo J, Yuan L, Chen Y, Chen M, Zeng M. 2023. Domination of an emerging erythromycin-resistant ptxP3 Bordetella pertussis clone in Shanghai, China. Int J Antimicrob Agents 62:106835. doi:10.1016/j.ijantimicag.2023.106835 37127126

[13] Mai Q, Wen J, Luo Y, Guo J, Qin Y, Lai W, Deng W, Ji C, Mai R, Zheng M, Chen Z, Chen Y, Gu C, Guo L, Li H, Tang Y, Huang D, Luo M. 2025. Molecular epidemiology and increasing macrolide resistance of Bordetella pertussis isolates in Guangzhou, China. BMC Infect Dis 25:1152. doi:10.1186/s12879-025-11577-z 41013351 PMC12465671

[14] Xu Z, Wang Z, Luan Y, Li Y, Liu X, Peng X, Octavia S, Payne M, Lan R. 2019. Genomic epidemiology of erythromycin-resistant Bordetella pertussis in China. Emerg Microbes Infect 8:461–470. doi:10.1080/22221751.2019.1587315 30898080 PMC6455148

[15] Zomer A, Otsuka N, Hiramatsu Y, Kamachi K, Nishimura N, Ozaki T, Poolman J, Geurtsen J. 2018. Bordetella pertussis population dynamics and phylogeny in Japan after adoption of acellular pertussis vaccines. Microb Genom 4:e000180. doi:10.1099/mgen.0.000180 29771235 PMC5994715

[16] Yao K, Deng J, Ma X, Dai W, Chen Q, Zhou K, Ye J, Shi W, Wang H, Li D, Wang H, Wang J, Zhang J, Wu D, Xie G, Shen K, Zheng Y, Yang Y. 2020. The epidemic of erythromycin-resistant Bordetella pertussis with limited genome variation associated with pertussis resurgence in China. Expert Rev Vaccines 19:1093–1099. doi:10.1080/14760584.2020.1831916 33034224

[17] Wang C, Zhang H, Zhang Y, Xu L, Miao M, Yang H, Liu Y, He S, Pang L. 2021. Analysis of clinical characteristics of severe pertussis in infants and children: a retrospective study. BMC Pediatr 21:65. doi:10.1186/s12887-021-02507-4 33546645 PMC7863367

